# Supplementary material for: Genome-wide comparative analysis reveals human-mouse regulatory landscape and evolution
Source: BMC Genomics. 2015 Feb 14;16(1):87. doi: 10.1186/s12864-015-1245-6 (PMC4333152; doi:10.1186/s12864-015-1245-6)
Supplement: Additional file 1: — Supplemental Figures and Tables. [file 12864_2015_1245_MOESM1_ESM.pdf]

Supplementary material

Olgert Denas

September 24, 2014

List of Figures

1 Workflow of data and experiments. 3
2 The number of TFos for all human and mouse cell types mapped by each of the considered whole genome alignments 4
3 For each human celltype-factor pair we tested whether the SeqCons rate (of all TFos how many are SeqCons) is higher than expected by a Binomial test 5
4 For each mouse celltype-factor pair we tested whether the SeqCons rate (of all TFos how many are SeqCons) is higher than expected by a Binomial test 6
5 The distribution of *mouse* mappable TFos across *cell types* 7
6 The distribution of *human* mappable TFos across *cell types* 8
7 The distribution of *mouse* mappable TFos across *transcription factors* 9
8 The distribution of *human* mappable TFos across *transcription factors* 10
9 The distribution of *mouse* mappable TFos *nucleotides* across *cell types* 11
10 The distribution of *human* mappable TFos *nucleotides* across *cell types* 12
11 The distribution of *mouse* mappable TFos *nucleotides* across *transcription factors* . . 13
12 The distribution of *human* mappable TFos *nucleotides* across *transcription factors* . 14
13 For each celltype-factor pair we tested whether the (log) binding signal over FunctCons or FunctActive elements was significantly different from that over SeqCons elements 15
14 For each celltype-factor pair we tested whether the (log) binding signal over FunctCons or FunctActive elements was significantly different from that over SeqCons elements 16
15 Classification of mappable elements for all three analogous cell types 17
16 Classification of mappable elements for all three analogous cell types 18
17 The plot shows the number of FunctActive elements from a query assay as a function of the subset size 19
18 The plot shows the number of FunctActive elements from a query assay with respect to a subset of assays from the other species 20
19 We modeled the situation of a set of assays that have no co-association, thus overlap with an exclusive set of TFos on the other species 21
20 A mouse Rad21 occupancy site mapped on human chromosome 20. Mapping is guided by the human-mouse whole genome alignments which report 5 insertions in human. We classified this mouse TFos as FunctCons, as its mapped version in human overlapps with Rad21 occupancy sites in K562. 22

List of Tables

1 Peak signal statistics by peak classes 23
2 Analogous cells 23
3 Counts of mappable, functionally active, and total TFos. The table reports both the element count and the coverage in mega bases 24
4 Number of TFos mapped by each alignment for select assays. An element can be mapped if it overlaps DHS elements and shared human mouse DNA by half of its length. 24

5 Number of TFos mapped by each alignment for select assays. An element can be mapped if it overlaps DHS elements and shared human mouse DNA by half of its length. Continues 25
6 Number of TFos mapped by each alignment for select assays. An element can be mapped if it overlaps DHS elements and shared human mouse DNA by half of its length. 26

1 Supplementary figures

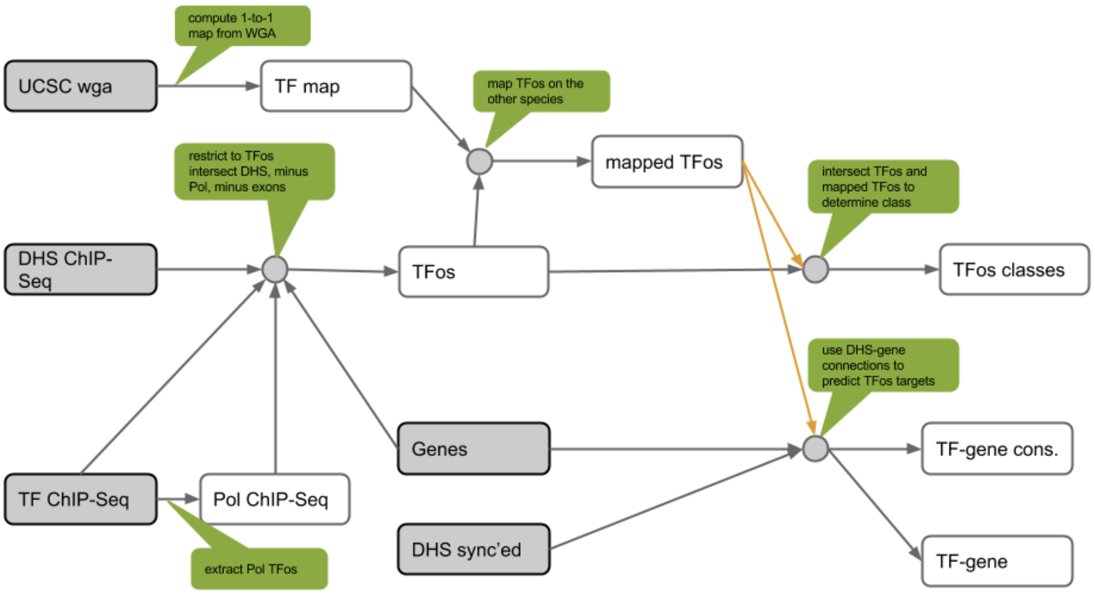

Figure 1: Workflow of data and experiments.

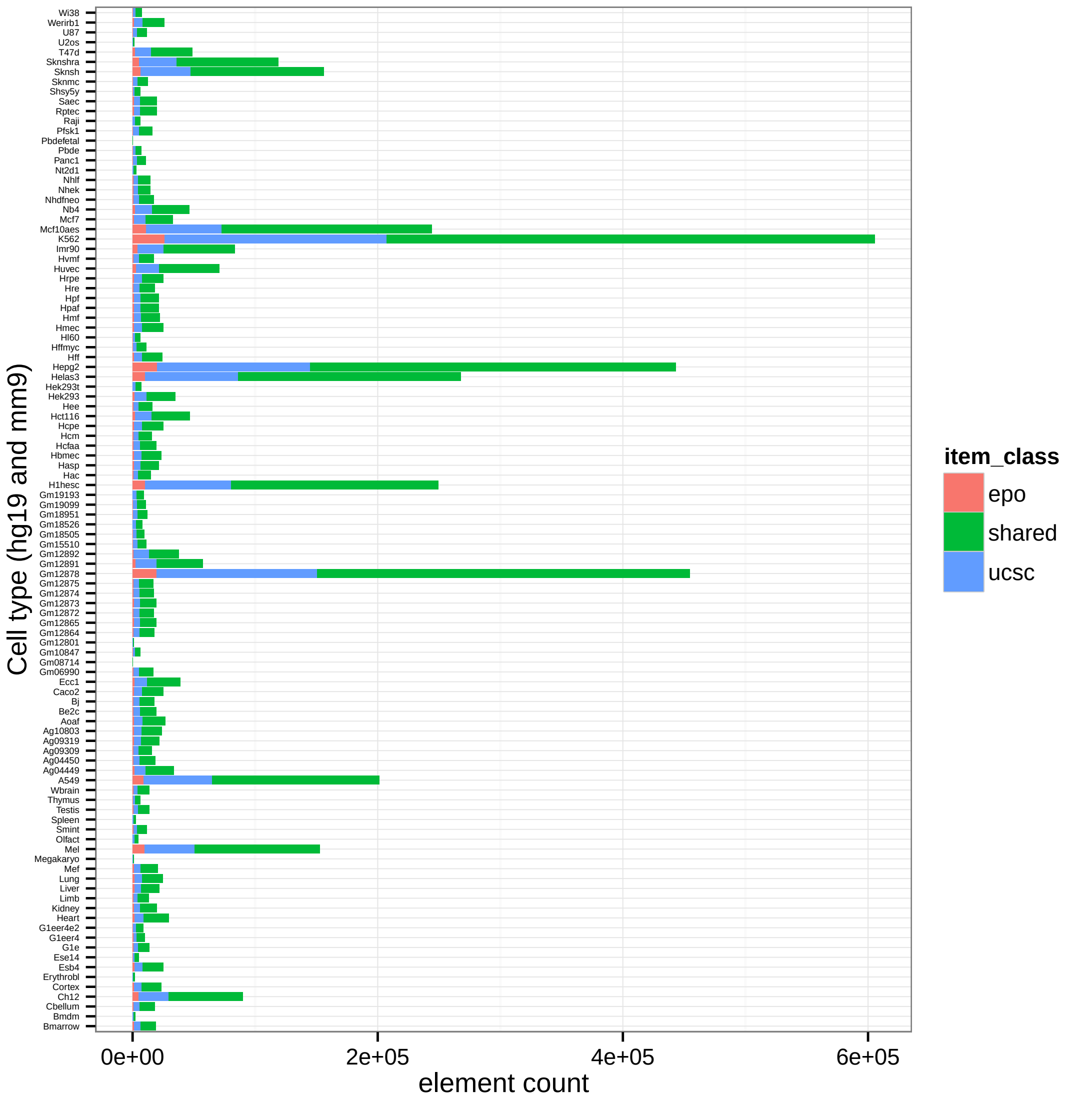

Figure 2: The number of TFos for all human and mouse cell types mapped by each of the considered whole genome alignments. Here we show, only those TFos that overlap by half their length with a DHS peak. See supplementary Table 4 for more details.

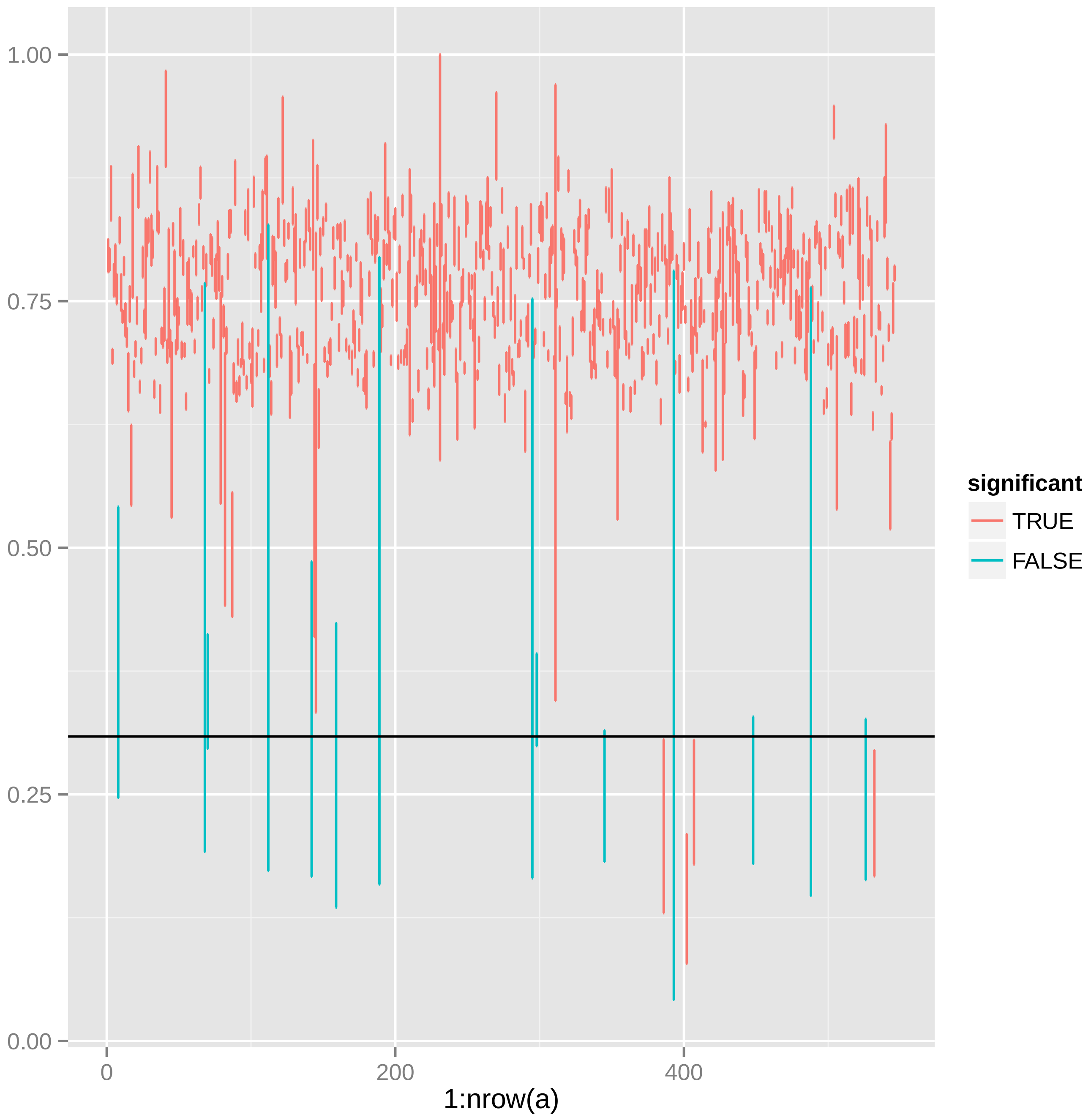

Figure 3: For each human celltype-factor pair we tested whether the SeqCons rate (of all TFos how many are SeqCons) is higher than expected by a Binomial test. The vertical lines show the estimated 99% confidence interval and the black line indicates the expected rate. The color of the vertical lines indicates whether the interval contains the expected value.

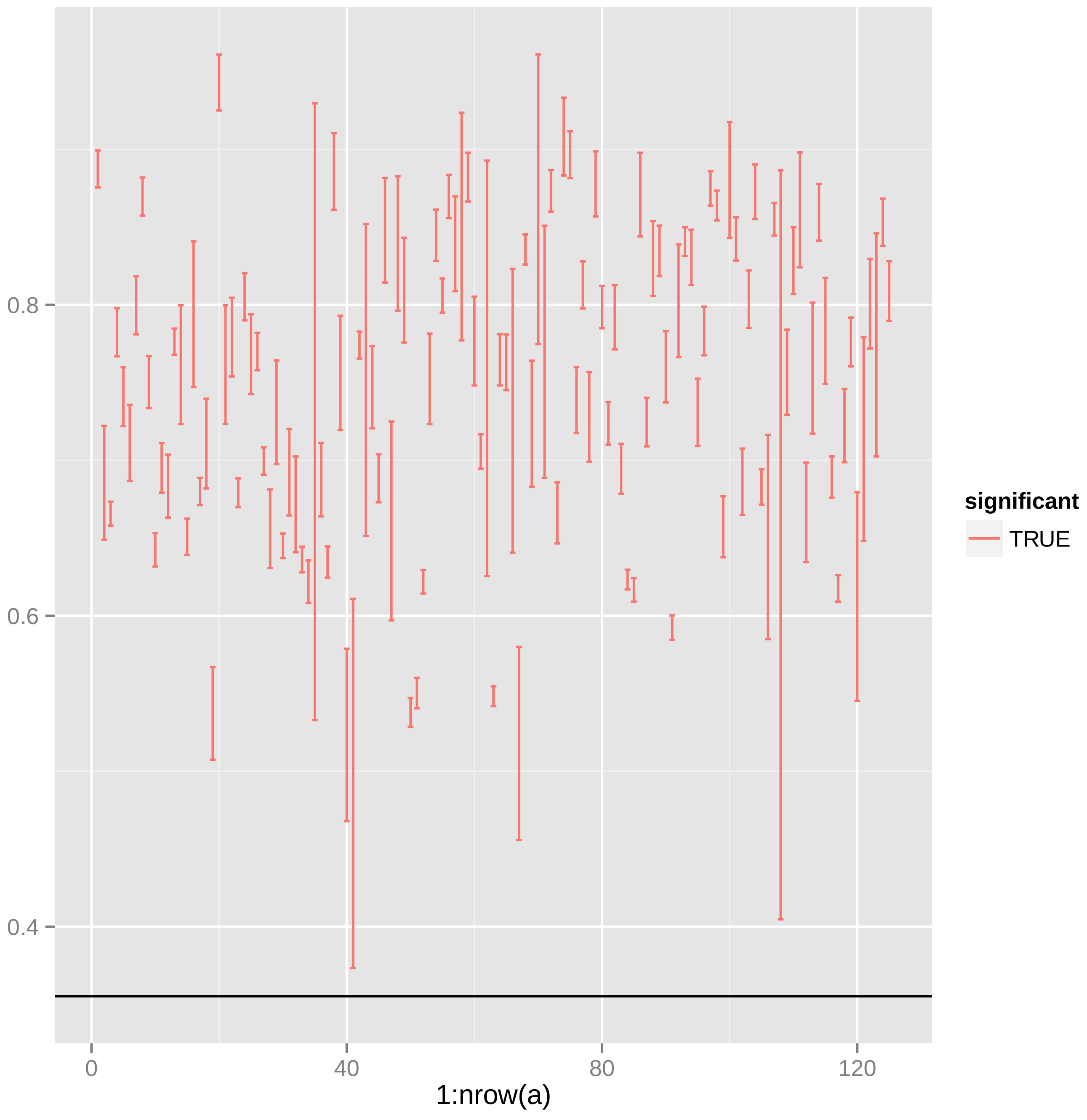

Figure 4: For each mouse celltype-factor pair we tested whether the SeqCons rate (of all TFos how many are SeqCons) is higher than expected by a Binomial test. The vertical lines show the estimated 99% confidence interval and the black line indicates the expected rate. The color of the vertical lines indicates whether the interval contains the expected value.

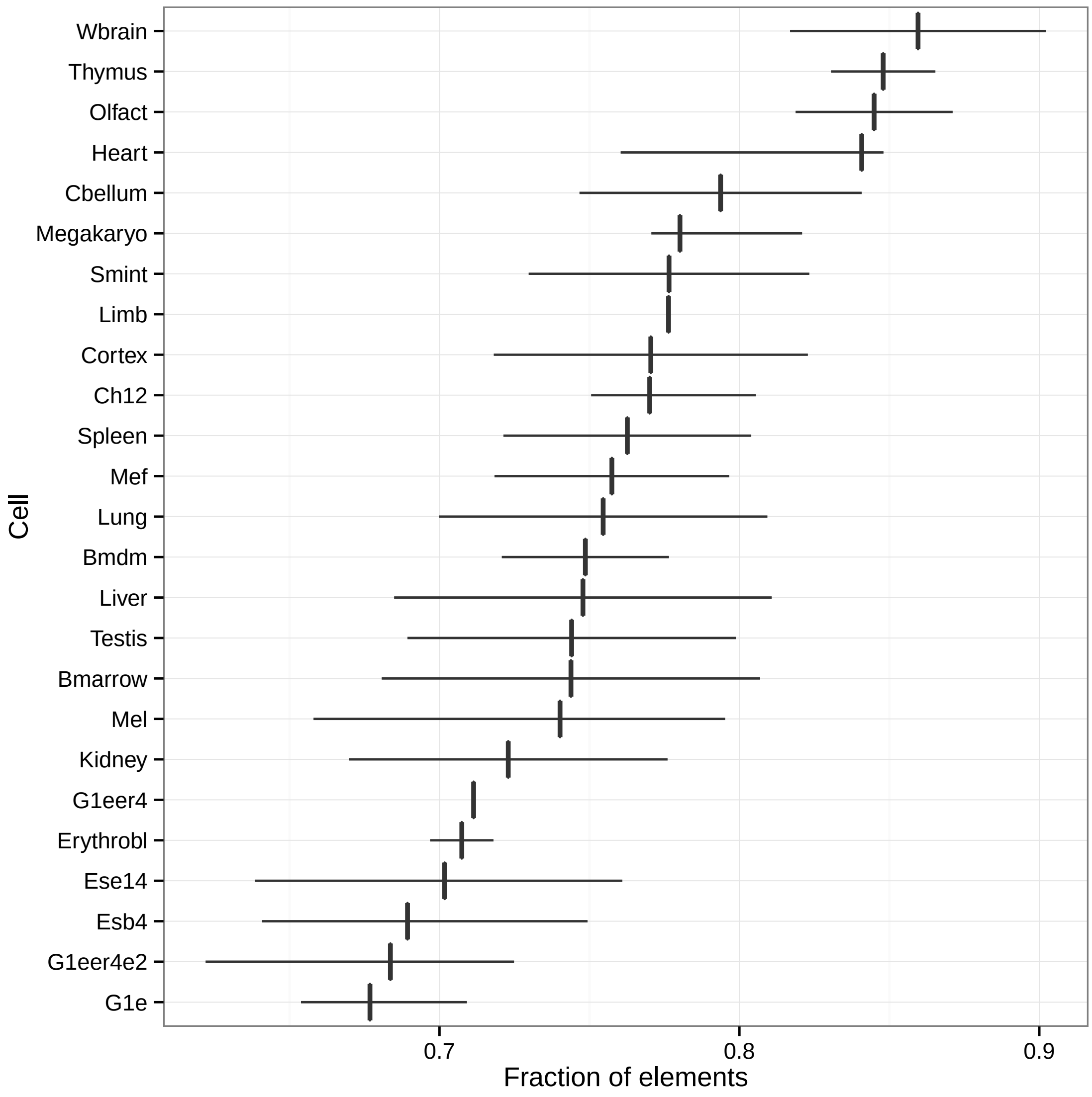

Figure 5: The distribution of *mouse* mappable TFos across *cell types*. The box-plot for each cell type summarizes the distribution of values for the fraction of elements that can be mapped on the other species. Occupied segments for each cell type contribute one value to the distribution.

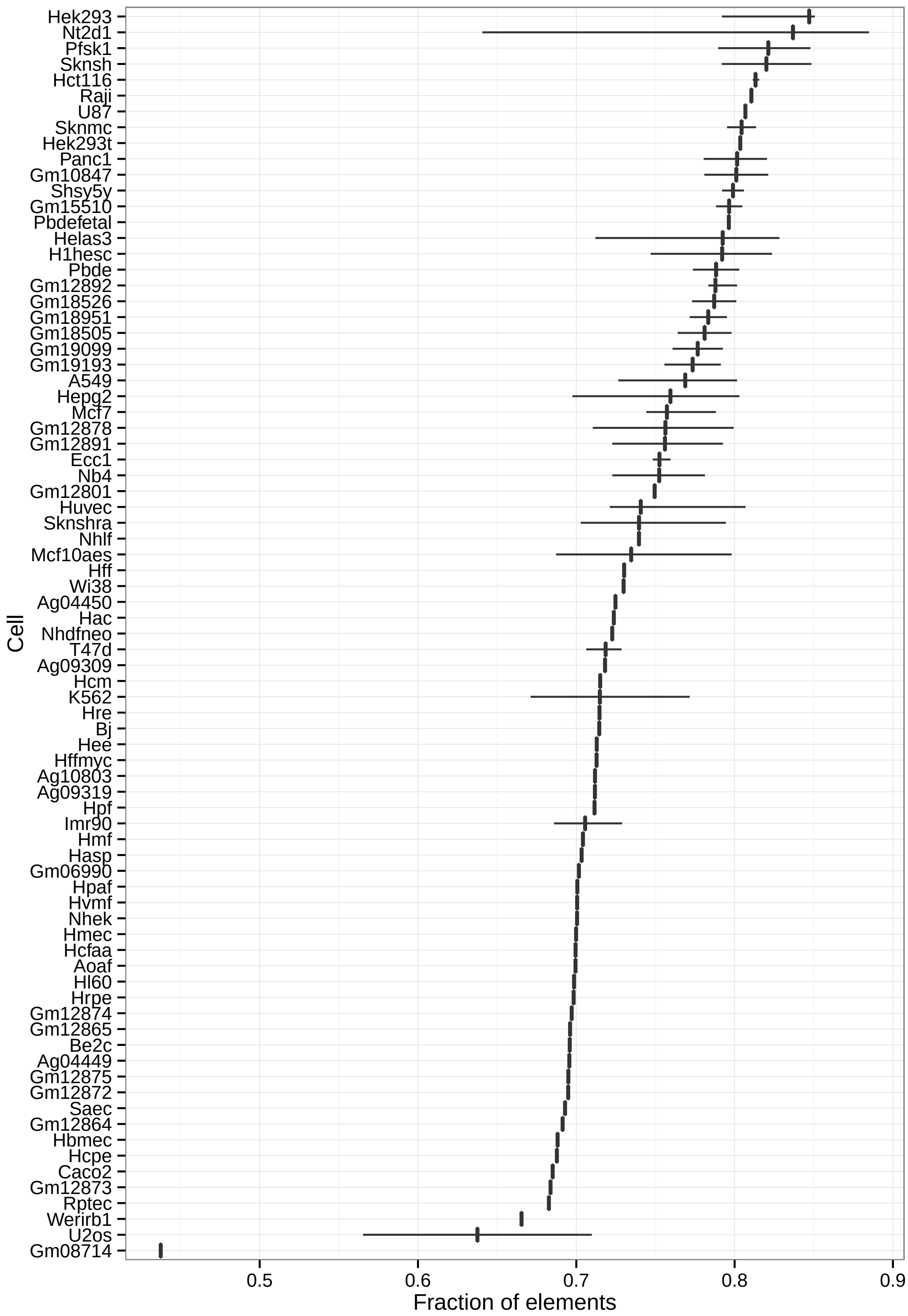

Figure 6: The distribution of *human* mappable TFos across *cell types*. The box-plot for each cell type summarizes the distribution of values for the fraction of elements that can be mapped on the other species.

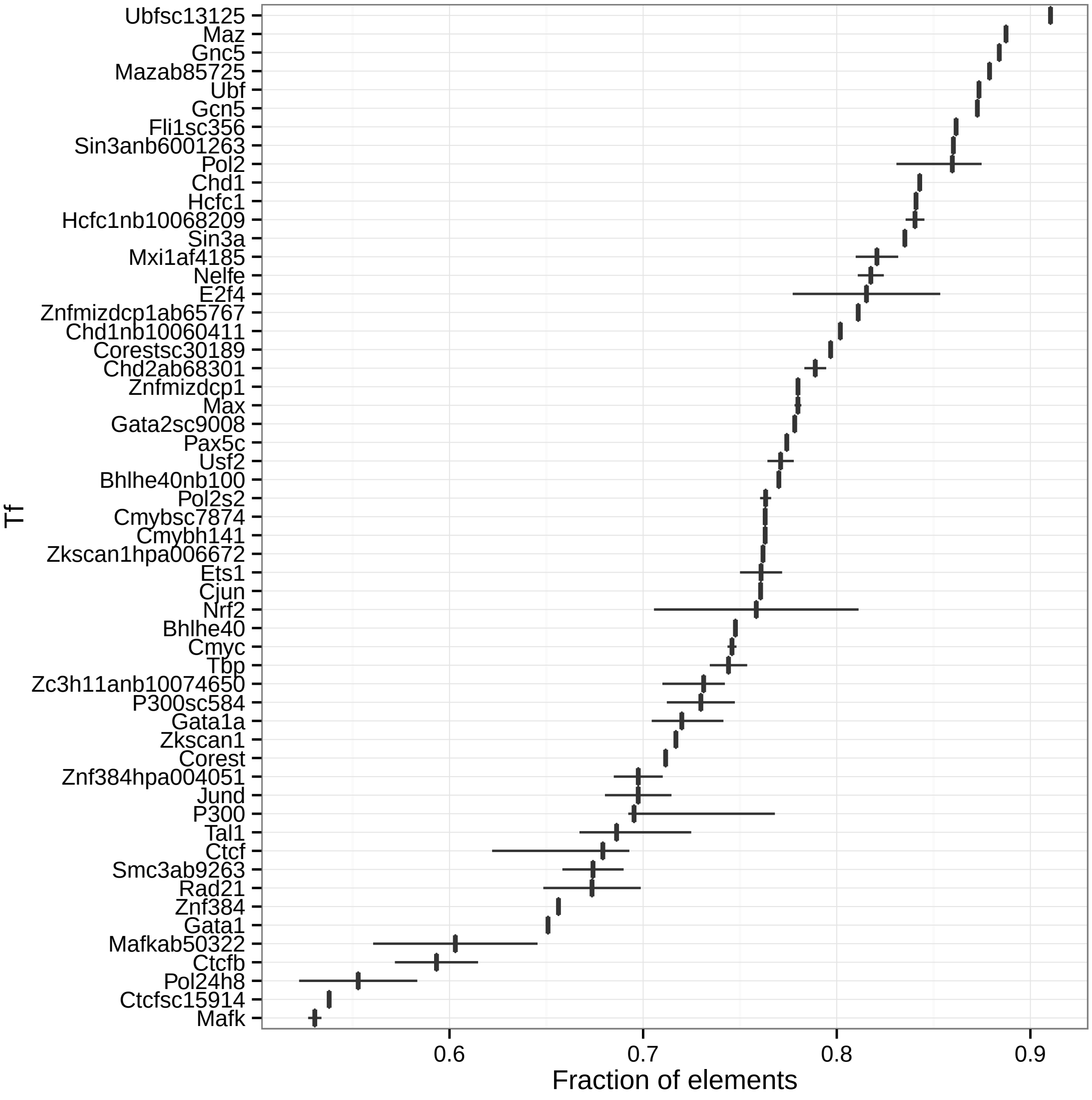

Figure 7: The distribution of *mouse* mappable TFos across *transcription factors*. The box-plot for each transcription factor summarizes the distribution of values for the fraction of elements that can be mapped on the other species.

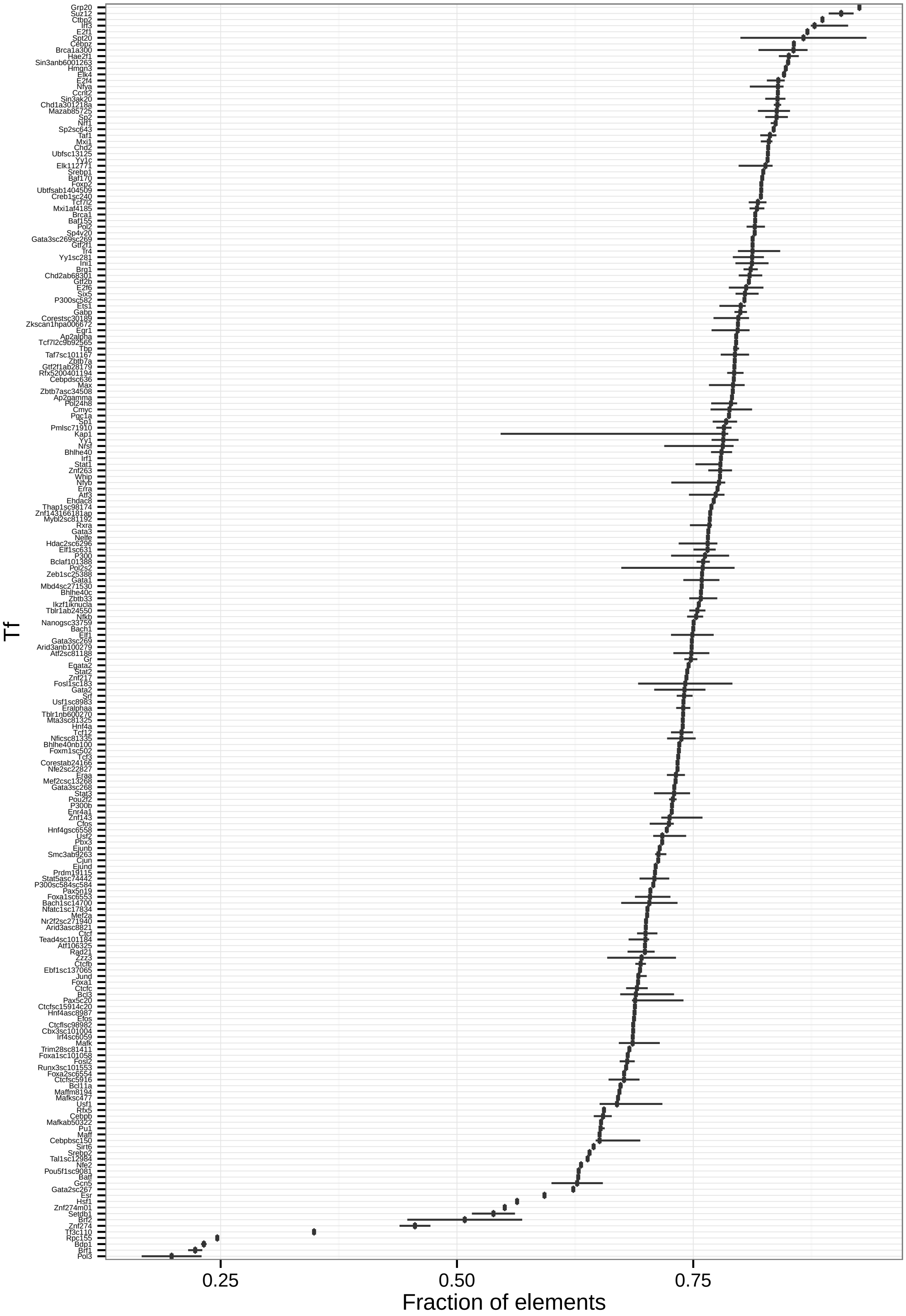

Figure 8: The distribution of *human* mappable TFos across *transcription factors*. The box-plot for each transcription factor summarizes the distribution of values for the fraction of elements that can be mapped on the other species.

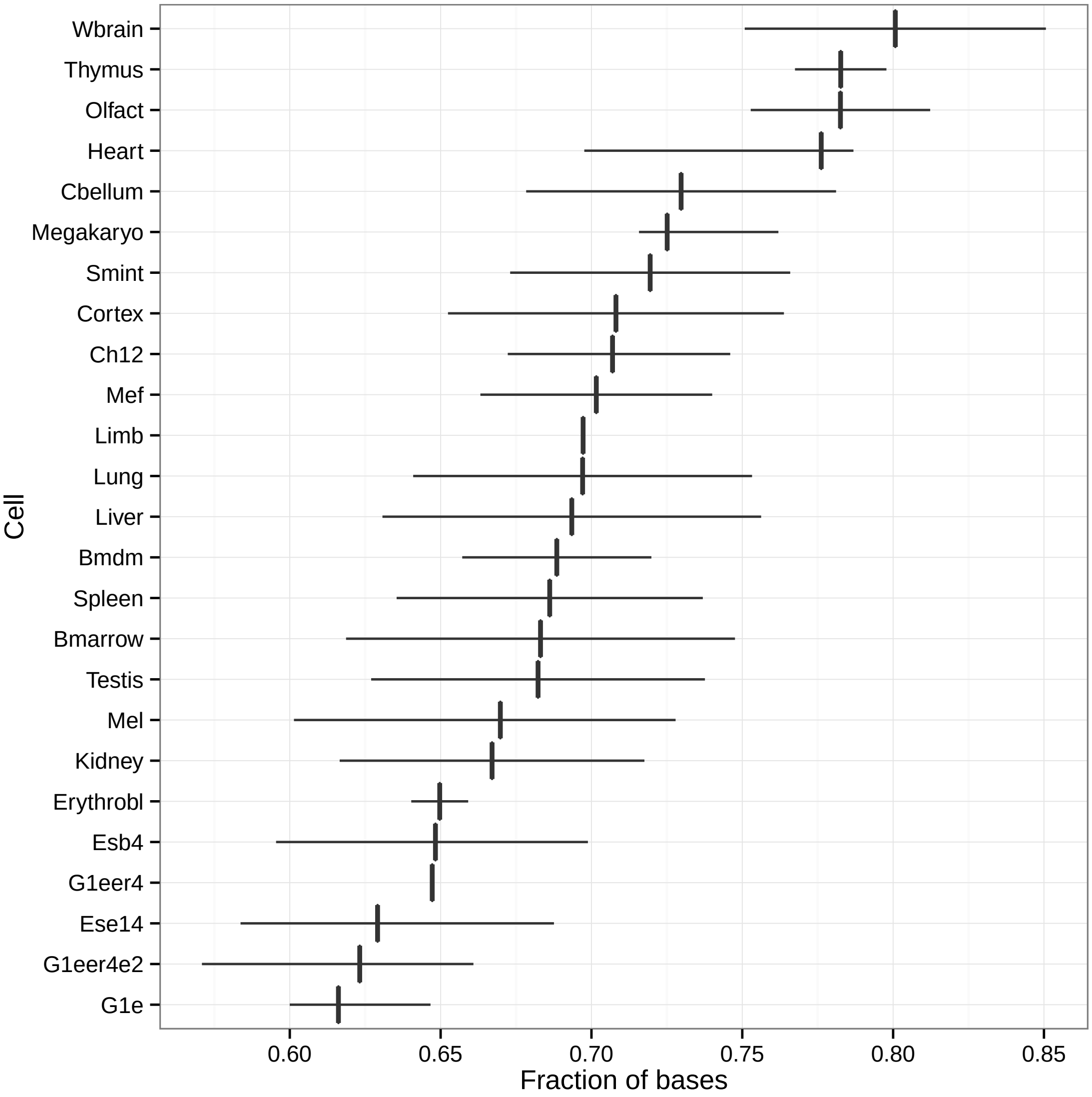

Figure 9: The distribution of *mouse* mappable TFos *nucleotides* across *cell types*. The box-plot for each cell type summarizes the distribution of values for the fraction of nucleotides that can be mapped on the other species.

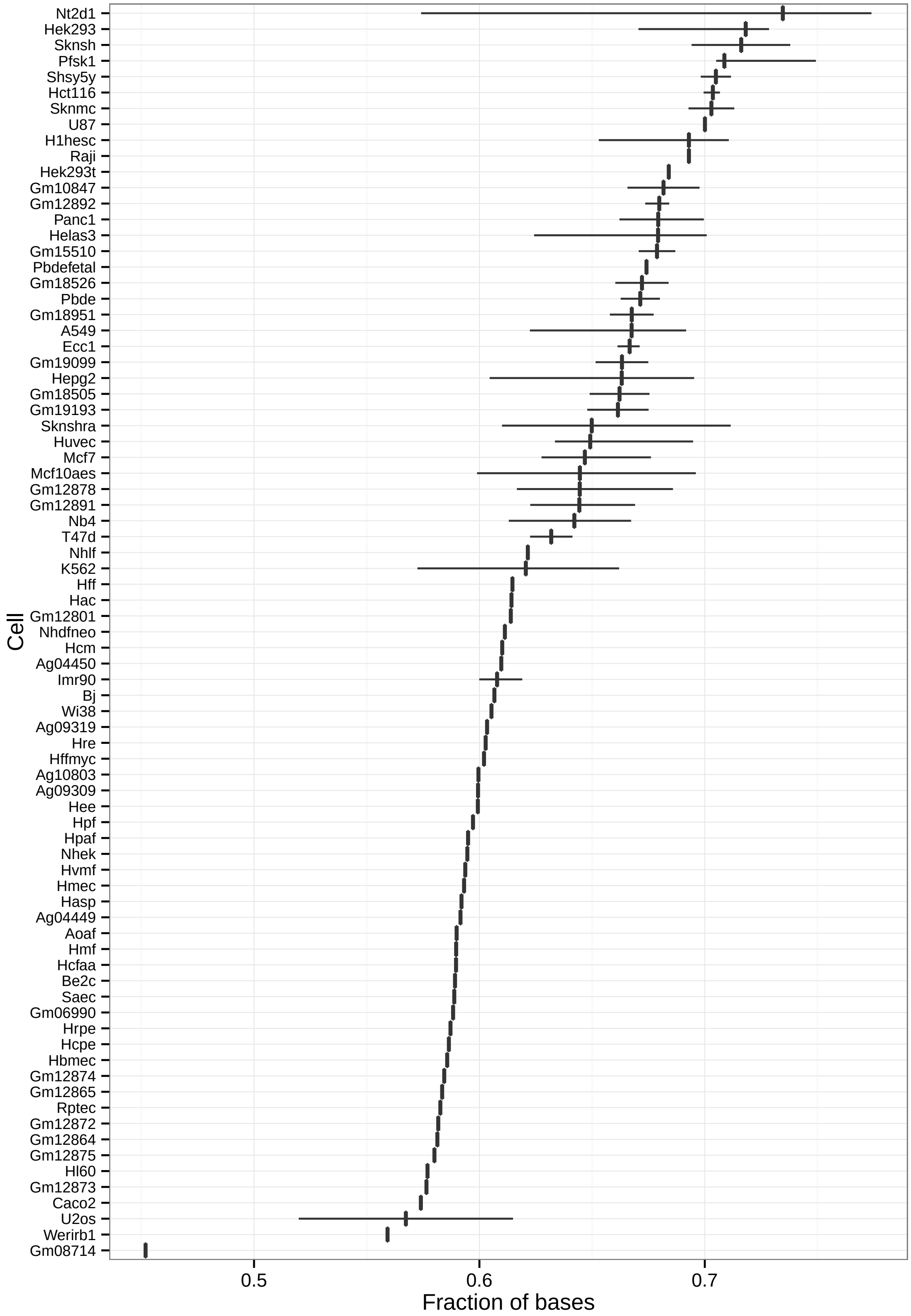

Figure 10: The distribution of *human* mappable TFos *nucleotides* across *cell types*. The box-plot for each cell type summarizes the distribution of values for the fraction of nucleotides that can be mapped on the other species.

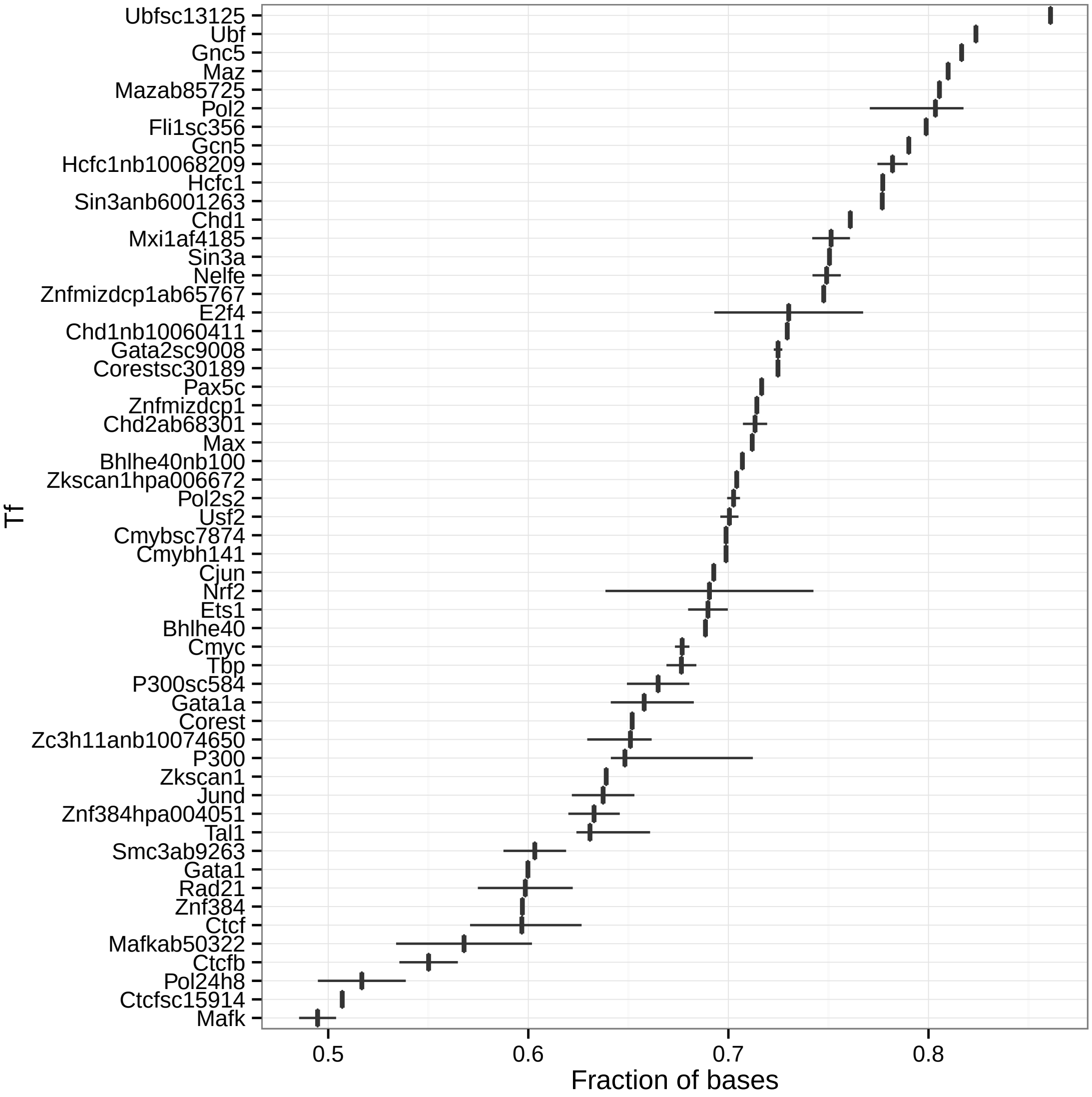

Figure 11: The distribution of *mouse* mappable TFos *nucleotides* across *transcription factors*. The box-plot for each cell type summarizes the distribution of values for the fraction of nucleotides that can be mapped on the other species.

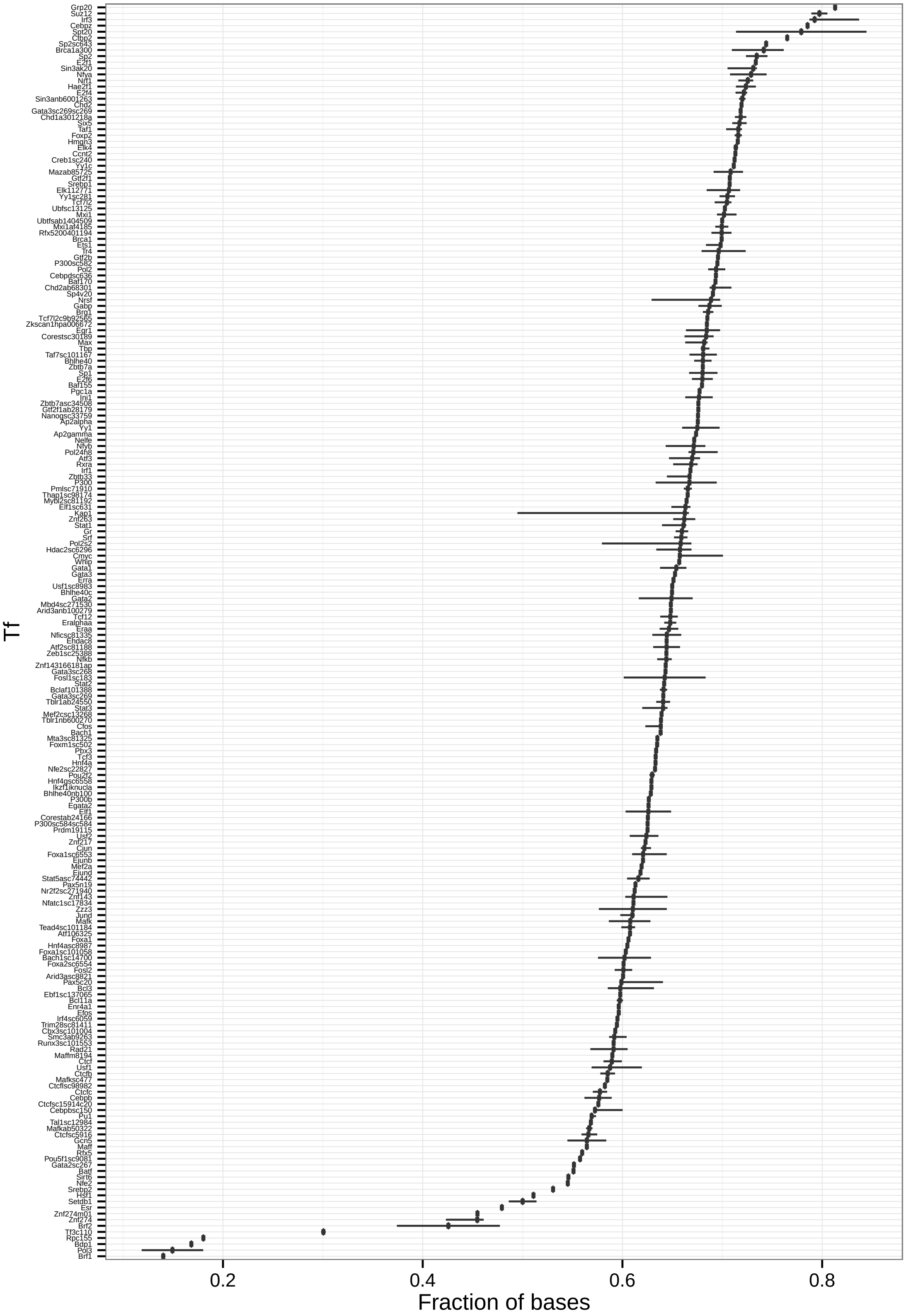

Figure 12: The distribution of *human* mappable TFos *nucleotides* across *transcription factors*. The box-plot for each cell type summarizes the distribution of values for the fraction of nucleotides that can be mapped on the other species.

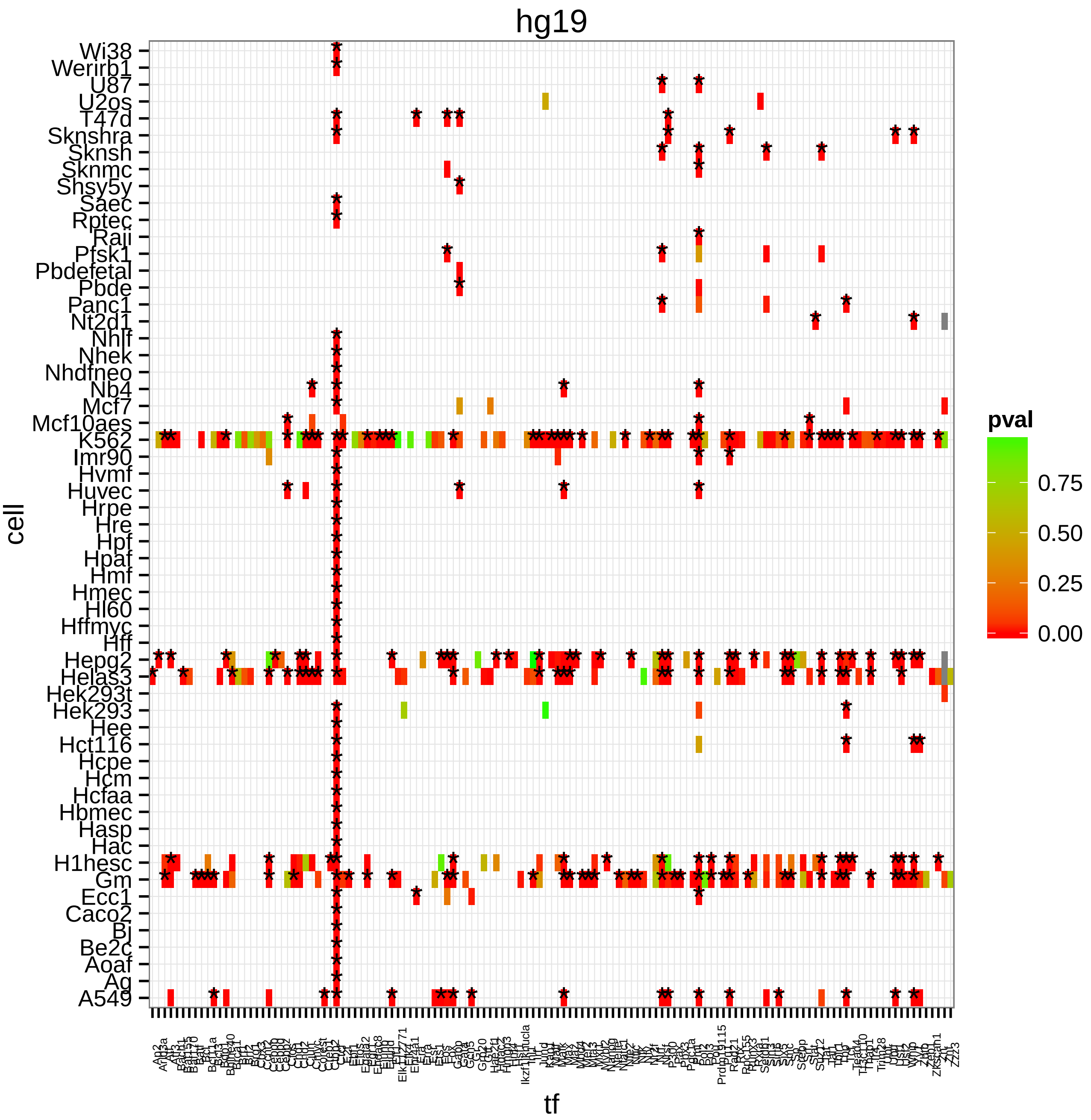

Figure 13: For each celltype-factor pair we tested whether the (log) binding signal over FunctCons or FunctActive elements was significantly different from that over SeqCons elements. Cases with a Bonferroni corrected error rate of 1% are marked with a '*'; those for which there were not enough elements to perform the test are labeled with gray; white positions are missing data.

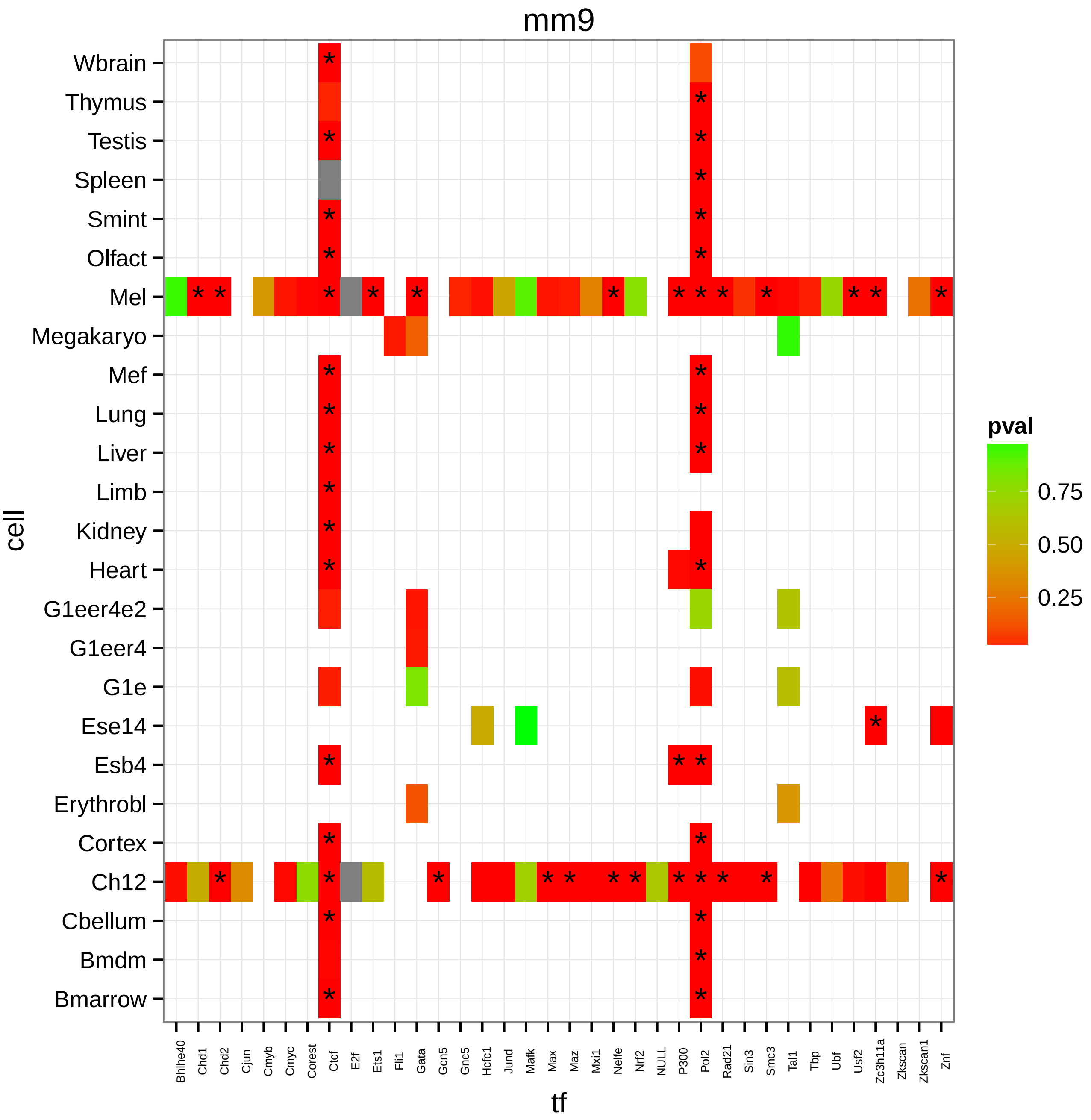

Figure 14: For each celltype-factor pair we tested whether the (log) binding signal over FunctCons or FunctActive elements was significantly different from that over SeqCons elements. Cases with a Bonferroni corrected error rate of 1% are marked with a '*'; those for which there were not enough elements to perform the test are labeled with gray; white positions are missing data.

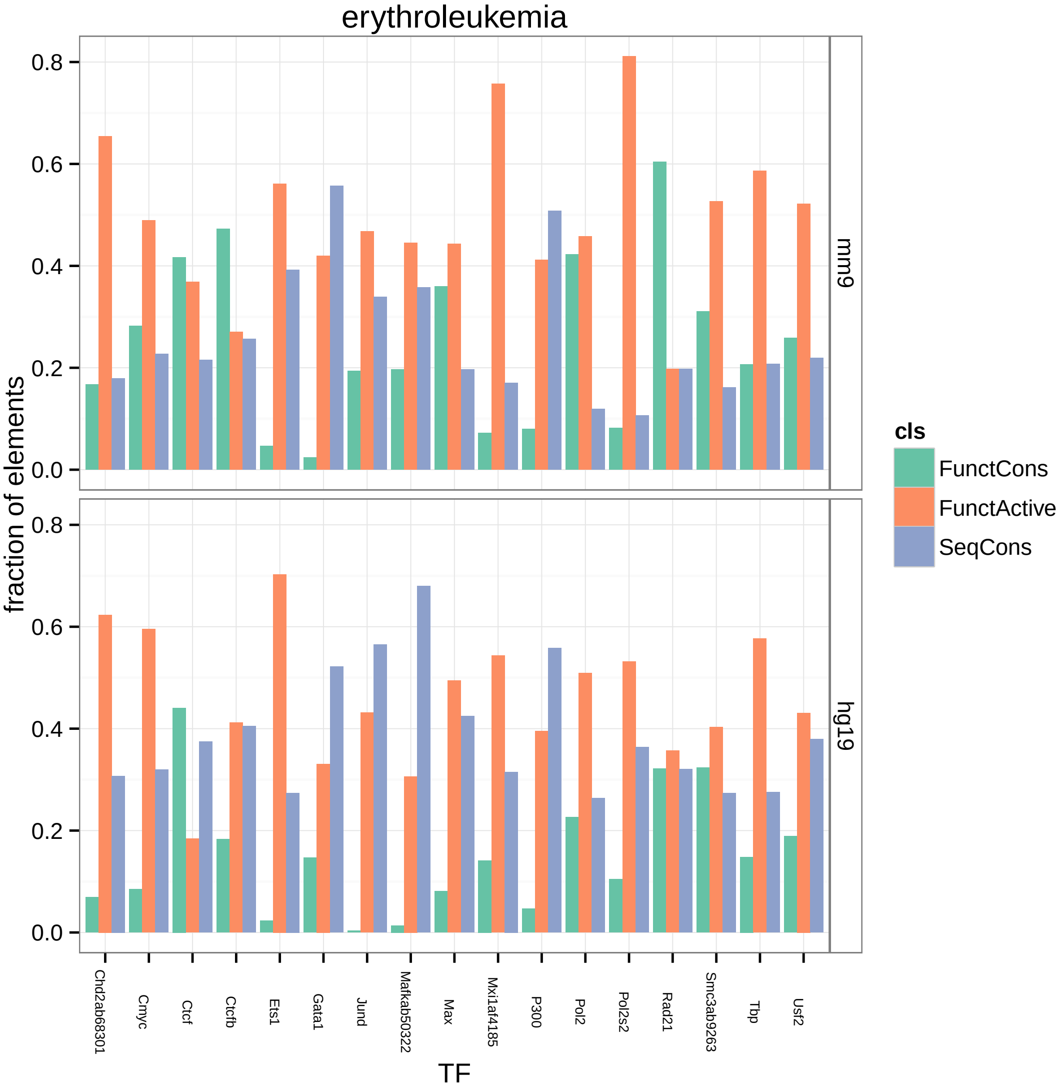

Figure 15: Classification of mappable elements for all three analogous cell types

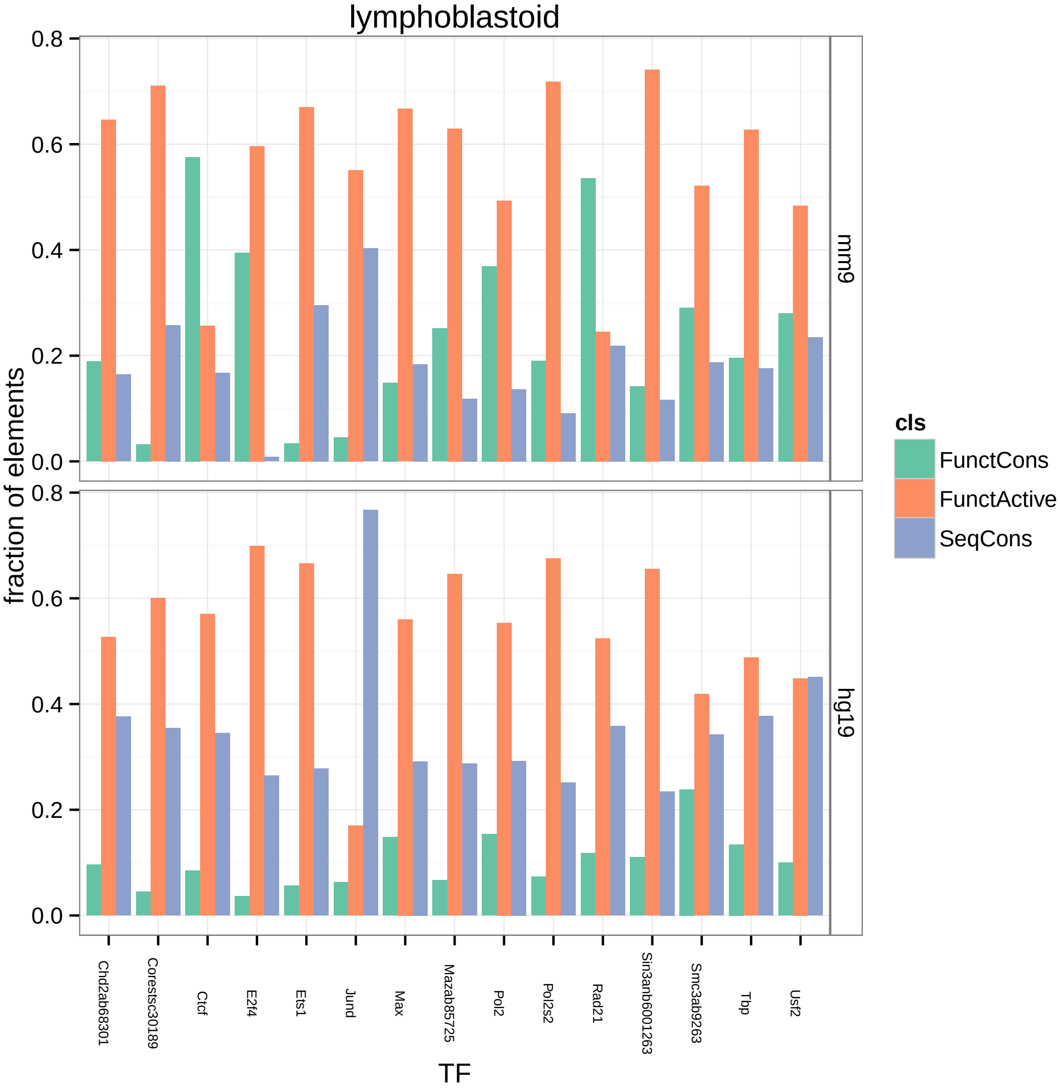

Figure 16: Classification of mappable elements for all three analogous cell types

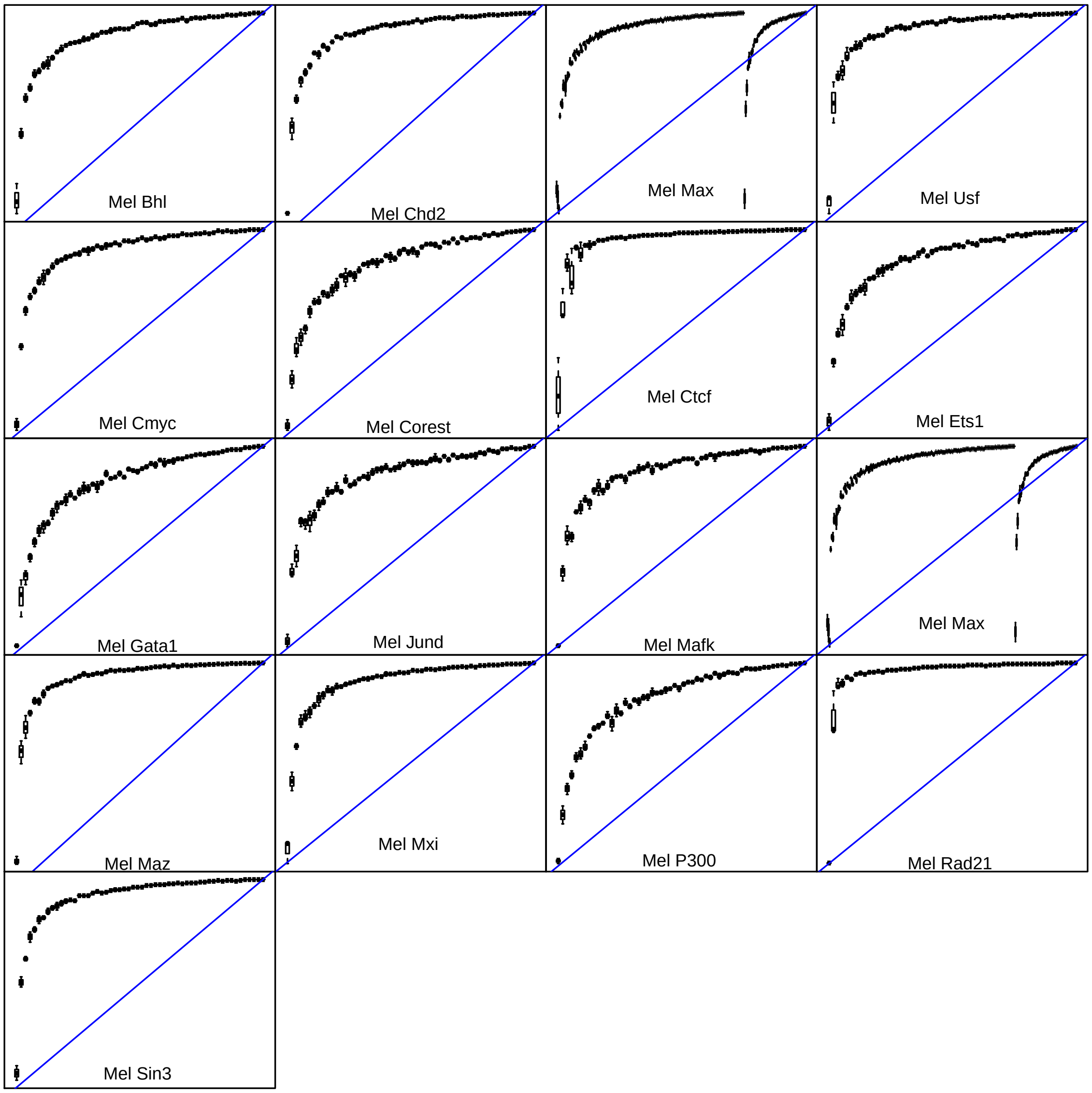

Figure 17: The plot shows the number of FunctActive elements from a query assay as a function of the subset size. We perform multiple countings for each number of assays from the other species

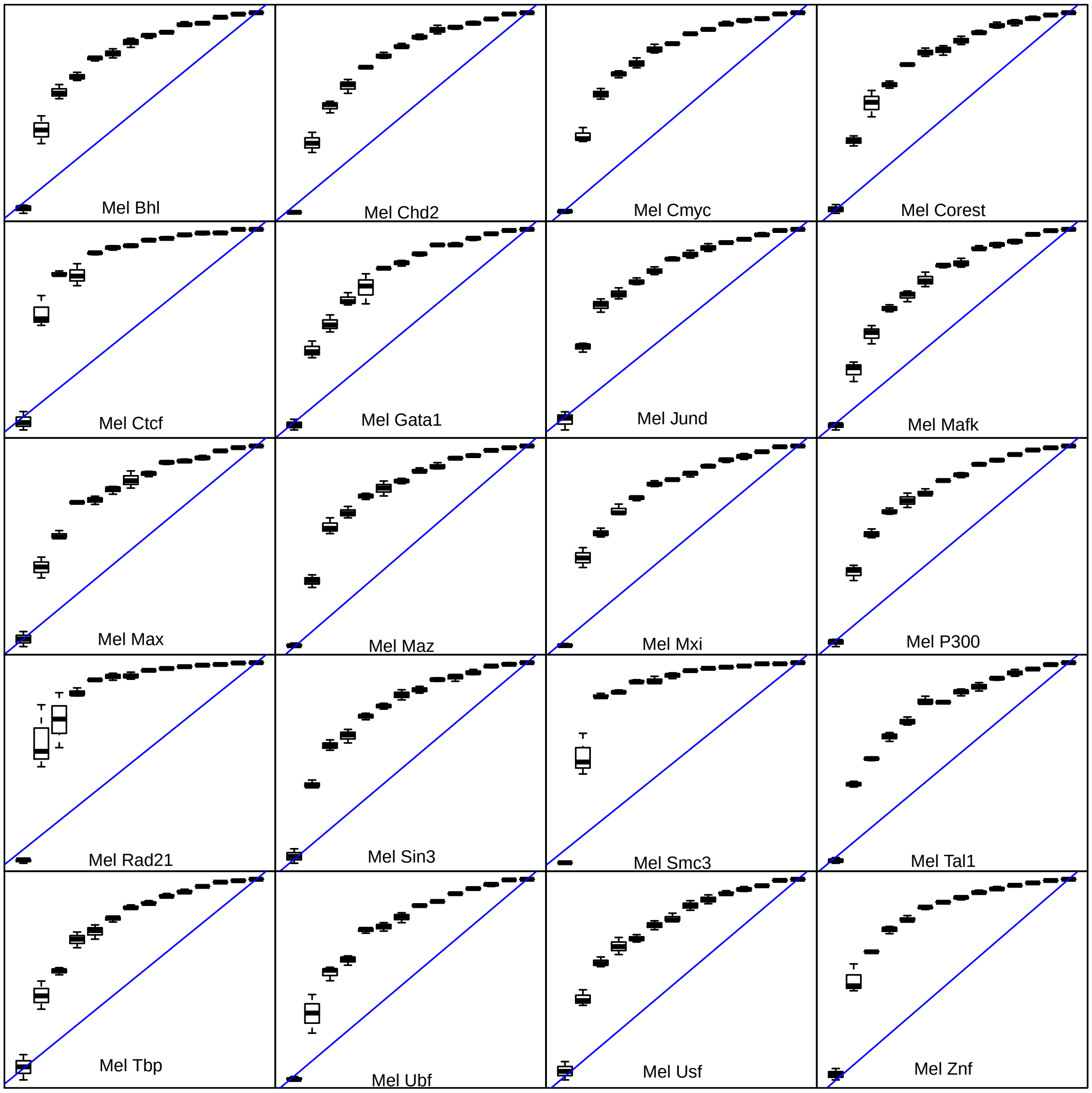

Figure 18: The plot shows the number of FunctActive elements from a query assay with respect to a subset of assays from the other species. We perform multiple countings for each number of assays from the other species

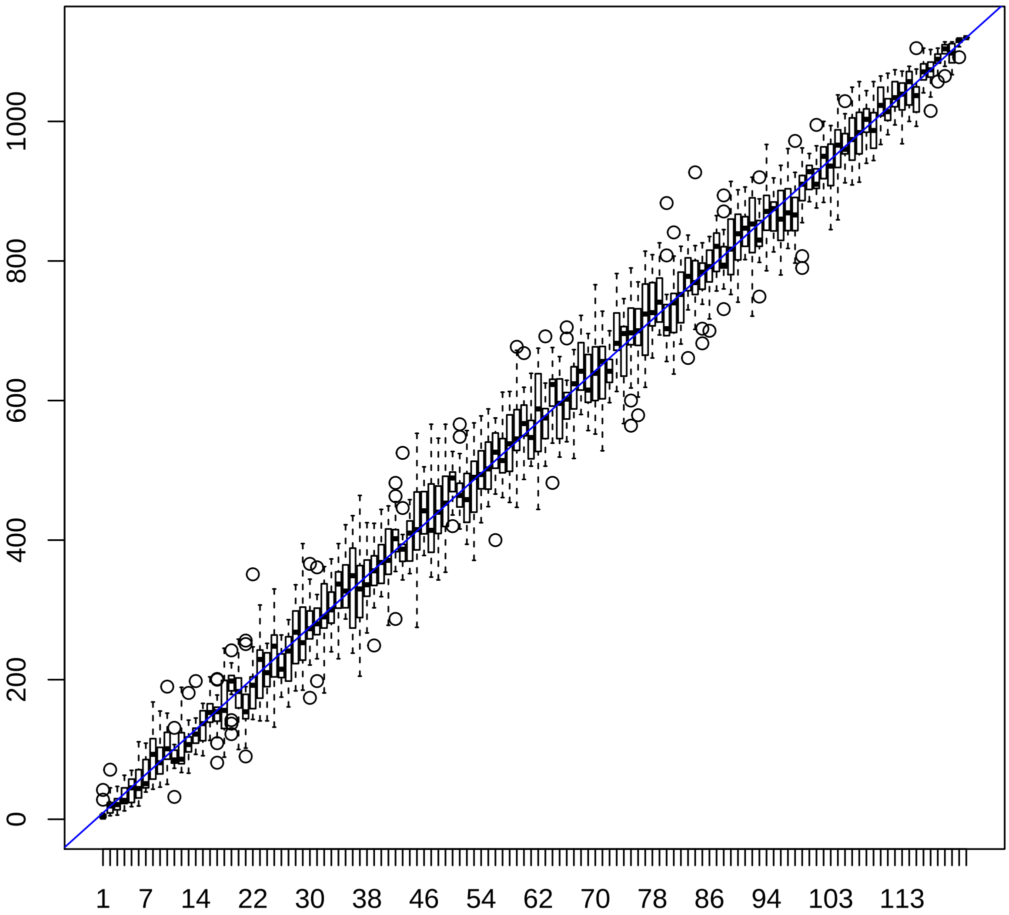

Figure 19: We modeled the situation of a set of assays that have no co-association, thus overlap with an exclusive set of TFos on the other species. The figure shows a simulation for 100 assays covergin 1000 TFos on the other species such that the sets of covered TFos are pairwise disjoint. We perform multiple countings for each number of assays from the other species.

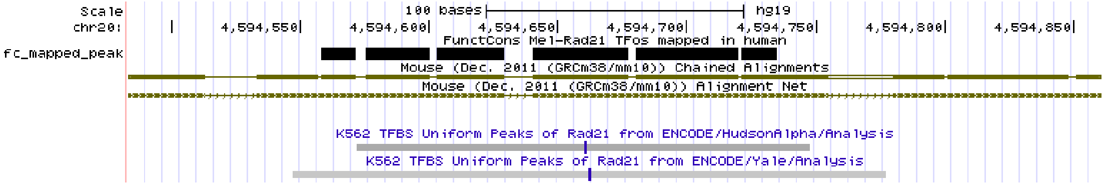

Figure 20: A mouse Rad21 occupancy site mapped on human chromosome 20. Mapping is guided by the human-mouse whole genome alignments which report 5 insertions in human. We classified this mouse TFos as FunctCons, as its mapped version in human overlapps with Rad21 occupancy sites in K562.

2 Suplementary tables

	Min.	X1st.Qu.	Median	Mean	X3rd.Qu.	Max.
FunctActive (mm9)	5.4	38.3	74.0	131.1	164.5	7960.0
FunctCons (mm9)	14.6	74.9	153.7	227.4	302.5	6875.0
NonMapped (mm9)	5.4	41.2	83.1	135.2	174.9	8899.0
SeqCons (mm9)	5.4	38.5	74.0	121.2	157.0	4958.0
FunctActive (hg19)	9.8	34.7	65.9	98.4	137.9	1370.0
FunctCons (hg19)	14.1	41.3	77.9	98.6	139.1	829.9
NonMapped (hg19)	9.8	33.1	59.5	85.6	114.1	1588.0
SeqCons (hg19)	9.7	30.9	51.0	74.9	95.8	1239.0

Table 1: Peak signal statistics by peak classes

	Analogous cell pair (human, mouse)	Transcription factor
1	leuk (K562, Mel)	Gata1
2	leuk (K562, Mel)	Pol2s2
3	leuk (K562, Mel)	Usf2
4	leuk (K562, Mel)	Ctcfb
5	leuk (K562, Mel)	Ets1
6	leuk (K562, Mel)	Tbp
7	leuk (K562, Mel)	Max
8	leuk (K562, Mel)	Mafkab50322
9	leuk (K562, Mel)	Chd2ab68301
10	leuk (K562, Mel)	P300
11	leuk (K562, Mel)	Jund
12	leuk (K562, Mel)	Smc3ab9263
13	leuk (K562, Mel)	Mxi1af4185
14	leuk (K562, Mel)	Ctcf
15	leuk (K562, Mel)	Cmyc
16	leuk (K562, Mel)	Rad21
17	leuk (K562, Mel)	Pol2
18	lymph (Gm12878, Ch12)	Pol2s2
19	lymph (Gm12878, Ch12)	Sin3anb6001263
20	lymph (Gm12878, Ch12)	Usf2
21	lymph (Gm12878, Ch12)	Ets1
22	lymph (Gm12878, Ch12)	Tbp
23	lymph (Gm12878, Ch12)	Max
24	lymph (Gm12878, Ch12)	Chd2ab68301
25	lymph (Gm12878, Ch12)	Jund
26	lymph (Gm12878, Ch12)	Smc3ab9263
27	lymph (Gm12878, Ch12)	Corestsc30189
28	lymph (Gm12878, Ch12)	Mazab85725
29	lymph (Gm12878, Ch12)	E2f4
30	lymph (Gm12878, Ch12)	Ctcf
31	lymph (Gm12878, Ch12)	Rad21
32	lymph (Gm12878, Ch12)	Pol2

Table 2: Analogous cells

	Species	Counting	Total Count	Function conserved	Sequence conserved
1	mm9	elements	727680	397846	503710
2	hg19	elements	5330864	2065660	3850414
3	mm9	Mega-nt	32	16	22
4	hg19	Mega-nt	121	25	83

Table 3: Counts of mappable, functionally active, and total TFos. The table reports both the element count and the coverage in mega bases

	cell	epo	ucsc	both	species
1	Cbellum	13339	16961	12301	mm9
2	Erythrobl	1472	1919	1370	mm9
3	Cortex	17450	21978	16072	mm9
4	Ch12	65349	84997	60454	mm9
5	Mel	111971	143033	102328	mm9
6	Bmarrow	13758	17662	12558	mm9
7	G1eer4e2	6610	8332	6007	mm9
8	Limb	10018	12634	9243	mm9
9	Heart	22077	28145	20571	mm9
10	G1eer4	7543	9520	6872	mm9
11	Ese14	3738	4795	3401	mm9
12	Wbrain	10163	12937	9416	mm9
13	Mef	15423	19776	14283	mm9
14	Lung	18262	23237	16796	mm9
15	Bmdm	1503	1944	1353	mm9
16	Testis	10113	12846	9209	mm9
17	Liver	16232	20546	14857	mm9
18	Spleen	1979	2770	1874	mm9
19	Smint	8595	11012	7880	mm9
20	Megakaryo	633	850	603	mm9
21	Thymus	4530	6123	4234	mm9
22	G1e	10300	13041	9353	mm9
23	Olfact	3385	4535	3198	mm9
24	Kidney	14969	18594	13616	mm9
25	Esb4	18483	23375	16845	mm9

Table 4: Number of TFos mapped by each alignment for select assays. An element can be mapped if it overlaps DHS elements and shared human mouse DNA by half of its length.

	cell	epo	ucsc	both	species
1	A549	145435	192624	136593	hg19
2	Helas3	191883	257474	181513	hg19
3	Gm12878	323618	434881	303925	hg19
4	U2os	1085	1529	1013	hg19
5	H1hesc	179399	238861	168954	hg19
6	Pbde	5011	7039	4773	hg19
7	Mcf10aes	182743	232988	171603	hg19
8	Gm12891	39863	54887	37533	hg19
9	Ecc1	28441	37530	26925	hg19
10	K562	424430	579307	398262	hg19
11	Hepg2	318167	423487	298333	hg19
12	Nb4	32771	44031	30571	hg19
13	Gm06990	12317	15951	11439	hg19
14	Huvec	51885	68268	49157	hg19
15	Gm12872	12756	16409	11787	hg19
16	Wi38	5526	7106	5123	hg19
17	T47d	35984	46540	33810	hg19
18	Mcf7	23622	31147	22094	hg19
19	Hcm	11542	14928	10751	hg19
20	Hasp	16039	20502	14901	hg19
21	Nhek	10680	13814	9941	hg19
22	Hpaf	16072	20530	14939	hg19
23	Hmf	16625	21272	15498	hg19
24	Hct116	33158	45045	31284	hg19
25	Pfsk1	11124	15420	10651	hg19
26	Gm12892	25328	36539	24239	hg19
27	Hek293	25114	33422	23552	hg19
28	Hl60	4840	6146	4455	hg19
29	Rptec	14744	18840	13675	hg19
30	Panc1	7341	10445	7004	hg19
31	Imr90	62159	79214	58040	hg19
32	Hee	12157	15463	11285	hg19
33	Caco2	18754	23826	17375	hg19
34	Hek293t	4898	6799	4695	hg19
35	Hffmyc	8425	10735	7806	hg19
36	Ag09319	16126	20650	15035	hg19
37	Sknshra	88506	113500	83167	hg19
38	Be2c	14342	18389	13315	hg19
39	Nhlf	10561	13521	9825	hg19
40	Sknsh	115218	149442	108746	hg19
41	Hrpe	18594	23642	17257	hg19
42	Gm15510	7589	10884	7242	hg19
43	Werirb1	19255	24568	17815	hg19
44	Gm12864	13074	16900	12118	hg19
45	Hbmec	17228	22036	15998	hg19
46	Saec	14583	18802	13537	hg19
47	Aoaf	19868	25343	18479	hg19
48	Gm18526	5204	7519	4981	hg19
49	Gm12865	14135	18284	13104	hg19
50	Gm18951	8262	11677	7868	hg19

Table 5: Number of TFos mapped by each alignment for select assays. An element can be mapped if it overlaps DHS elements and shared human mouse DNA by half of its length. Continues ...

	cell	epo	ucsc	both	species
51	Nt2d1	2431	3320	2382	hg19
52	Gm19099	7419	10467	7078	hg19
53	Gm18505	6578	9431	6282	hg19
54	Hmec	18667	24058	17411	hg19
55	Nhdfneo	12788	16358	11901	hg19
56	Sknmc	8670	11862	8255	hg19
57	Hvmf	12829	16426	11886	hg19
58	Bj	13007	16754	12095	hg19
59	Hpf	15975	20473	14891	hg19
60	Hcpe	18749	23982	17440	hg19
61	Raji	4356	6256	4145	hg19
62	Hac	11025	14109	10283	hg19
63	Ag04450	13815	17771	12862	hg19
64	Hcfaa	14202	18203	13216	hg19
65	Hre	13388	17115	12457	hg19
66	Gm10847	4313	6156	4121	hg19
67	Ag04449	24796	31787	23088	hg19
68	U87	8224	11328	7859	hg19
69	Ag10803	17574	22431	16350	hg19
70	Shsy5y	4937	6263	4720	hg19
71	Gm12875	12409	16123	11517	hg19
72	Gm19193	6284	9018	6028	hg19
73	Gm12801	862	1089	797	hg19
74	Gm08714	1	7	1	hg19
75	Pbdefetal	258	348	248	hg19
76	Gm12873	14378	18541	13320	hg19
77	Hff	17984	22979	16688	hg19
78	Gm12874	12534	16237	11610	hg19
79	Ag09309	11504	14748	10680	hg19

Table 6: Number of TFos mapped by each alignment for select assays. An element can be mapped if it overlaps DHS elements and shared human mouse DNA by half of its length.
